# Clinical and Radiological Outcomes of Oblique Lateral Lumbar Interbody Fusion

**DOI:** 10.7759/cureus.4029

**Published:** 2019-02-07

**Authors:** Ali Abbasi, Kamran Khaghany, Vali Orandi, Hamid Abbasi

**Affiliations:** 1 Internal Medicine, Pritzker School of Medicine, Chicago, USA; 2 Radiology, Lake Region Healthcare, Fergus Falls, USA; 3 Neurosurgery, Inspired Spine Health, Minneapolis, USA

**Keywords:** oblique lateral lumbar interbody fusion, ollif, degenerative disk surgery

## Abstract

Oblique lateral lumbar interbody fusion (OLLIF) is a novel operation for fusions of the lumbar spine from T12–S1. In OLLIF, the disk is approached from an oblique lateral angle guided by electrophysiological monitoring and biplanar fluoroscopy; the disk space is accessed through Kambin’s triangle. We present perioperative, clinical, patient-reported and radiological outcomes from a series of 303 OLLIF procedures on 568 levels performed by the same surgeon. For a single-level OLLIF, mean surgery time was 56.6 ± 37.7 minutes, with a blood loss of 42.2 ± 31.1 mL, fluoroscopy time of 198.8 ± 87.2 seconds and a hospital stay of 2.2 ± 1.7 days. At the one-year follow-up, 10-point pain scale scores improved from 8.6 ± 1.3 to 4.1 ± 3.0 (p < 0.001). Total Oswestry disability index score improved from 56.6% ± 15.3% to 38.6% ± 21.4% (p < 0.001). At the one-year follow-up, 15 (5%) patients had mild nerve root irritation defined as sensory symptoms and motor weakness better than 4/5. Only one patient had neuropraxia due to weakness (3/5). There was one case (0.3%) of superficial wound infection and one case of bleeding into the psoas major. Reoperation within one year was performed for 14 (4.7%) patients. Interbody fusion was achieved in 98.7% of levels. While OLLIF has previously been described, this study is the first to present clinical, patient-reported, and radiological outcomes of OLLIF. Review of the literature shows that OLLIF produces perioperative outcomes, complication rates, and fusion rates that compare favorably with similar procedures. We establish that OLLIF is a safe, efficient and efficacious procedure for fusions of the lumbar spine.

## Introduction

Low back pain is the most common source of years lived with disability worldwide and has a point prevalence of up to 30% in the United States [1]. Low back pain is commonly caused by degenerative changes to the spine, including the intervertebral disk and facet joints. Degenerative disc and facet disease leads to progressive disability by causing disc herniation and nerve entrapment, and changes at a single level often lead to a multilevel disease [2]. Rates of pain and disability related to degenerative spine disease have increased dramatically in recent years. As the prevalence of degenerative spine disease has increased, demand for surgical treatments and the associated costs have skyrocketed [1,3]. Considering these realities, advances in the surgical treatment of disc disorders have the potential to improve outcomes for millions of patients and significantly reduce overall health care costs.

Lumber interbody fusions are the most common surgical treatment for degenerative disk and spine disease. Posterior lumbar interbody fusion (PLIF) was developed in the 1950s and became the standard technique to achieve interbody fusion [4]. Transforaminal lumbar interbody fusion (TLIF) was first described in 1982 and quickly emerged as a popular alternative to PLIF because TLIF enables a unilateral approach and reduced surgical morbidity compared to PLIF, facilitating a faster recovery [5]. However, both TLIF and PLIF are open procedures that require the surgeon to strip muscles and supportive connective tissue from the spine during approach which may cause significant surgical morbidity and adjacent level disease [6].

Recently, multiple minimally invasive (MI) approaches for lumbar spine fusion have been developed. Posterior approaches include minimally invasive TLIF (MI-TLIF) [7] which has been shown to reduce blood loss and complication rates relative to open TLIF. MI-TLIF is essentially the same procedure as TLIF, but performed through a smaller surgical corridor, making it a technically challenging procedure that often increases surgery time relative to open TLIF. Additionally, MI-TLIF still disrupts posterior muscles and soft tissues that stabilize the spine, resulting in similar long-term outcomes as open TLIF [8]. To minimize the disruption of posterior structures, MI anterior and lateral approaches have been developed. Examples of anterior approaches include oblique lumbar interbody fusion anterior to psoas, where the approach is through the retroperitoneum for L2–L5 and through the peritoneum for L5–S1, with the patient in the lateral position [9]. This technique has shown some promise but results in increased urological and vascular complications [10]. Alternative techniques involve a lateral transpsoas approach, including extreme lateral interbody fusion, direct lateral interbody fusion (DLIF) and lumbar lateral interbody fusion. These techniques increase the likelihood of lumbar plexus and psoas muscle damage and may not be suited for accessing levels L4–S1 [11]. Due to these limitations, no MI spinal fusion technique has gained widespread popularity to date.

Oblique lateral lumbar interbody fusion (OLLIF) is a recent innovation in MI spinal fusion [12]. OLLIF is performed with the patient in the prone position and employs an oblique lateral approach that enables the instrumentation to pass through Kambin’s triangle, which is defined as the space between the exiting nerve, the superior border of the caudal vertebra, and the superior articulating process of the inferior facet. While it has long been shown that arthroscopic fusions through Kambin’s triangle are feasible [13], these procedures have suffered from high complication rates due to nerve damage [14]. To avoid these complications, we have modified the procedure by using electrophysiological monitoring and biplanar fluoroscopy to ensure a safe approach. Unlike other MI approaches to the lumbar spine, OLLIF can safely be employed from T12–S1 and does not require any ostomies. Although L5–S1 can be more difficult to approach, a more lateral approach called minimally invasive direct lateral interbody fusion (MIS-DLIF) can routinely be performed for L5–S1 with the same instruments and operating room setup [15]. Previous studies have shown that OLLIF is a safe and efficacious procedure for fusions of the lumbar spine and significantly reduces surgery time, hospital stay and blood loss relative to open TLIF [12]. However, detailed clinical and radiological outcomes for OLLIF have not previously been presented.

Here, we present a series of 303 consecutive OLLIF cases in a single surgeon, multiple hospital study. We collected perioperative data, complication rates, patient-reported outcomes, and imaging with a one-year follow-up.

## Materials and methods

Study design

This study is a retrospective case series including 303 OLLIF procedures performed by the same surgeon. Procedures were performed in five Minnesota hospitals. Institutional review board (IRB) exemption was granted by Pearl Pathways IRB on 30 January 2017 (IRB study number 17-TRIS-106). The Clinical Trial registration for this study is registered on clinicaltrials.gov as trial NCT03726190.

All patients underwent a full course of conservative therapy before being considered candidates for surgery. Conservative therapy included physical therapy, therapeutic injections, bracing and behavioral modification. Preoperative imaging included magnetic resonance imaging, X-ray of the lumbar spine with flexion and extension and, in many cases, a discogram and computed tomography (CT) scan. OLLIF is indicated for severe degenerative disc disease, spondylolisthesis, spinal stenosis and disc herniation. The following anatomical factors were relative contraindications for OLLIF: bony obstruction, significant spinal canal stenosis, large facet hypertrophy and grade II listhesis. Grade II spondylolisthesis is also technically more challenging, but can effectively be treated with the MIS-DLIF technique [15]. OLLIF has been used to correct scoliosis [16], but for this study, we excluded any patients with Cobb angles > 10°. Demographics of the study population along with indications for surgery are displayed in Table 1.

**Table 1:** Study group characteristics. Obesity is defined as Class I if BMI ≥ 30 and BMI < 35, Class II if BMI ≥ 35 and BMI < 40, Class III if BMI ≥ 40. Surgical levels indicate total number of fusions performed on each level. Many patients had multiple preoperative diagnoses. BMI: Body mass index; SD: Standard deviation.

Study group characteristics	Value count (%) or mean (SD)
n	303
Age (years) (mean (SD))	58 (16)
BMI (mean (SD))	31.16 (6.44)
Class I Obesity (n (%))	80 (26.5)
Class II Obesity (n (%))	54 (17.9)
Class III Obesity (n (%))	32 (10.6)
Number of levels (mean (SD))	1.87 (0.77)
L1-L2 ((n (%))	22 (7.3)
L2-L3 (n (%))	61 (20.1)
L3-L4 (n (%))	117 (38.6)
L4-L5 (n (%))	212 (70.0)
L5-S1 (n (%))	152 (50.2)
Preoperative Diagnosis	
Degenerative Disk Disease (n (%))	219 (72.3)
Herniated Disk (n (%))	106 (35.0)
Spondylolisthesis (n (%))	100 (33.0)
Spinal Stenosis (n (%))	51 (16.8)

The OLLIF procedure

**Figure 1:**
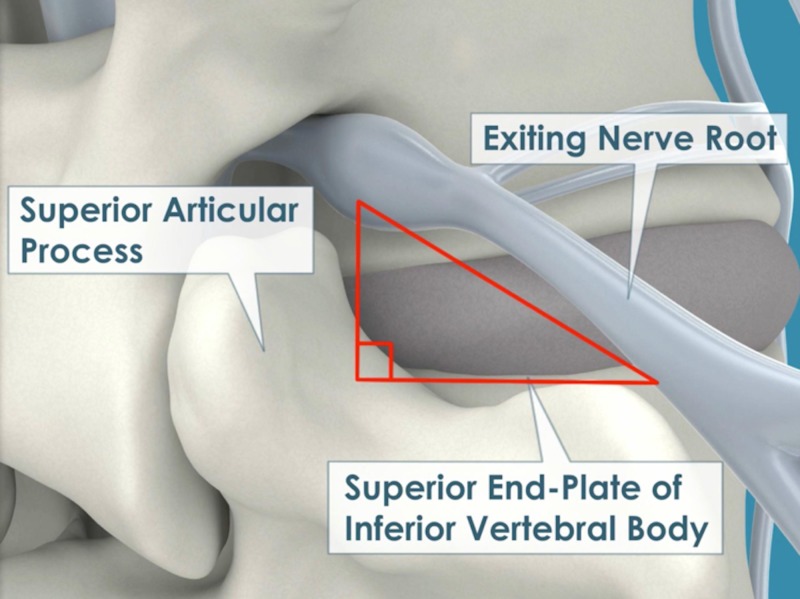
The disk is accessed through Kambin’s triangle, which is located between the exiting nerve root, the superior articular process and the superior end-plate of the inferior vertebral body.

**Figure 2:**
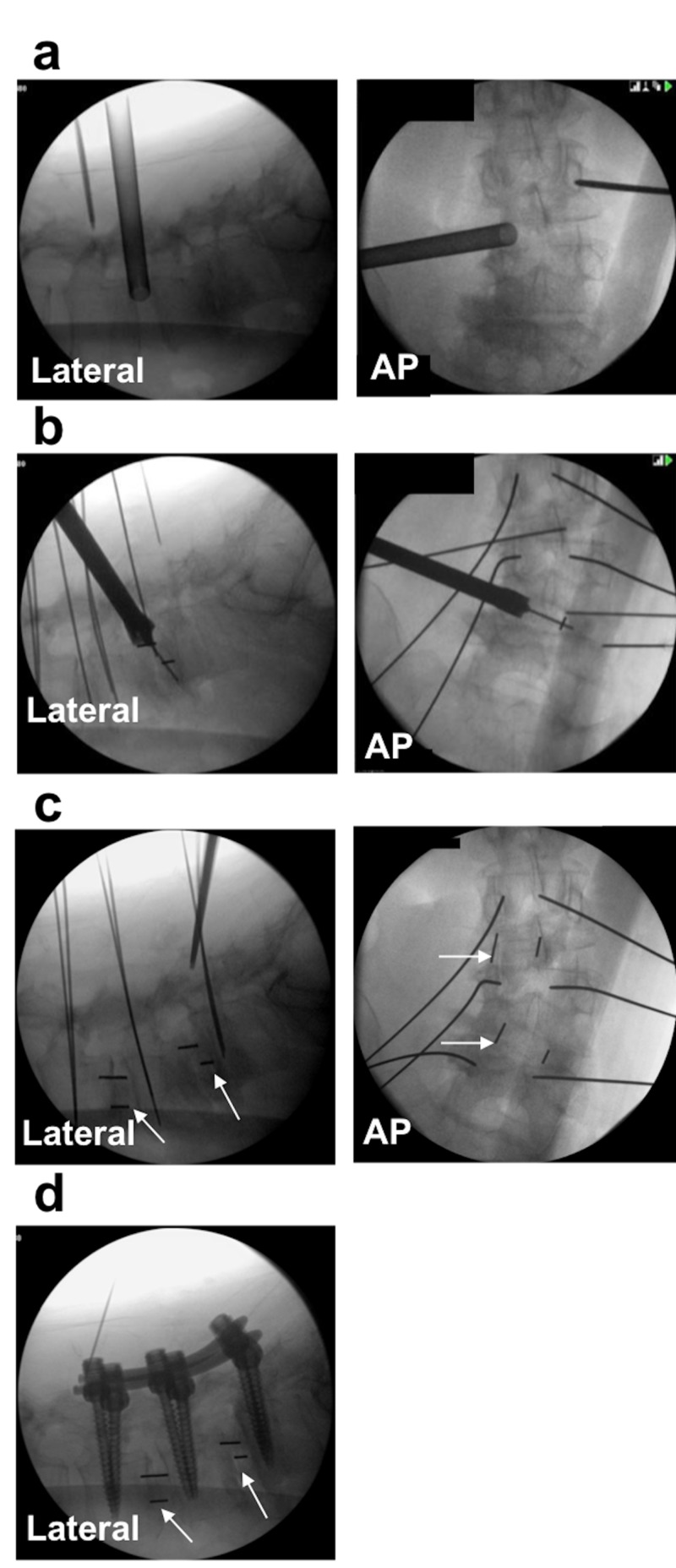
OLLIF viewed under fluoroscopy. (a) The disc is approached guided by fluoroscopy and electrophysiological monitoring. (b) The cage is inserted. (c) The interbody fusion is complete; arrows indicate the location of cages. (d) OLLIF is complemented with percutaneous posterior pedicle screw fixation. OLLIF: Oblique lateral lumbar interbody fusion; AP: Anteroposterior.

We have previously described the technique for OLLIF in detail [12]. In brief, the patient is positioned on the operating table in the prone position and biplanar fluoroscopy is set up. The disk is approached at a 45° angle to the vertical plane, so that the instrumentation can pass through Kambin’s triangle (Figure 1). The disk is approached with a blunt probe (Figure 2a). Once the probe is stimulated up to our safety threshold of 4 mA, a dilator is inserted over the probe. Discectomy is performed through a 10 mm access portal. The cage is inserted under continued electrophysiological monitoring and fluoroscopy (Figure 2b). Finally, OLLIF is complemented with posterior pedicle screw fixation [17] (Figure 2c, 2d) to enable bilateral posterolateral fusion.

Outcome measures and analysis

Skin to skin surgery time, blood loss, fluoroscopy time, and hospital stay were recorded and entered into a custom database immediately after discharge. Because no suction is used in OLLIF, blood loss was measured by postoperatively weighing sponges and subtracting their dry weight. We also recorded wound infections and bleeding.

Patients underwent a physical examination and completed a modified Oswestry disability index (ODI) [18] before surgery and at the one-year follow-up, which was defined as having taken place at least 300 days after surgery to allow for flexible patient scheduling in a rural setting. Nerve deficits were classified as nerve root irritation if the patient exhibited dysesthesia, paresthesias or mild weakness of 4/5 or better. Deficits were classified as neuropraxia if the patient exhibited weakness of 3/5 or worse. Deficits were only categorized as complications if they were not present before surgery, appeared immediately after, and corresponded to the levels on which we operated.

We also obtained routine CT follow-up imaging to assess fusion and hardware failure at least 300 days after surgery. Images were read by two independent radiologists. Interbody fusion was defined as bony density crossing the disc space in two planes on standard CT scan with 2.5 mm cuts and 512 × 512 resolution with sagittal and coronal reconstruction images. We defined posterolateral (facet) fusion as bony density crossing the facet in one cut. Screw loosening was defined as a halo of more than 1 mm around the screw visualized in multiple cuts. Screw breach was defined as a screw protruding through the cortical bone of the pedicle by more than 2 mm. Bicortical screw placement was defined as a screw passing through the anterior or lateral border of the vertebral body by at least 2 mm.

We calculated summary statistics including mean and standard deviation for all measurements. We compared pre- and post-operative pain and Oswestry measurements by using paired T-Tests. We analyse the effects of body mass index (BMI) on surgery time by performing an ordinary least squares (OLS) linear regression estimating the effects of BMI and the number of surgical levels on surgery time. Data were collected in real time, placed in a custom database and exported for analysis and visualization in R3.4.

## Results

The study groups are outlined in Table 1. Patients were on average 58 ± 16 years old. More than half of all patients were obese, as 80 (26.5%) patients had Class I obesity, 54 (17.9%) patients had Class II obesity, and 33 (10.9%) patients had Class III obesity. The most common surgical level was L4–L5 with 70.0% of the procedures treating this level, followed by L5–S1 which was treated in 50.2% of the procedures. The most common preoperative diagnoses were degenerative disc disease (72.3% of patients), herniated disc (35%), and spondylolisthesis (33%).

**Figure 3:**
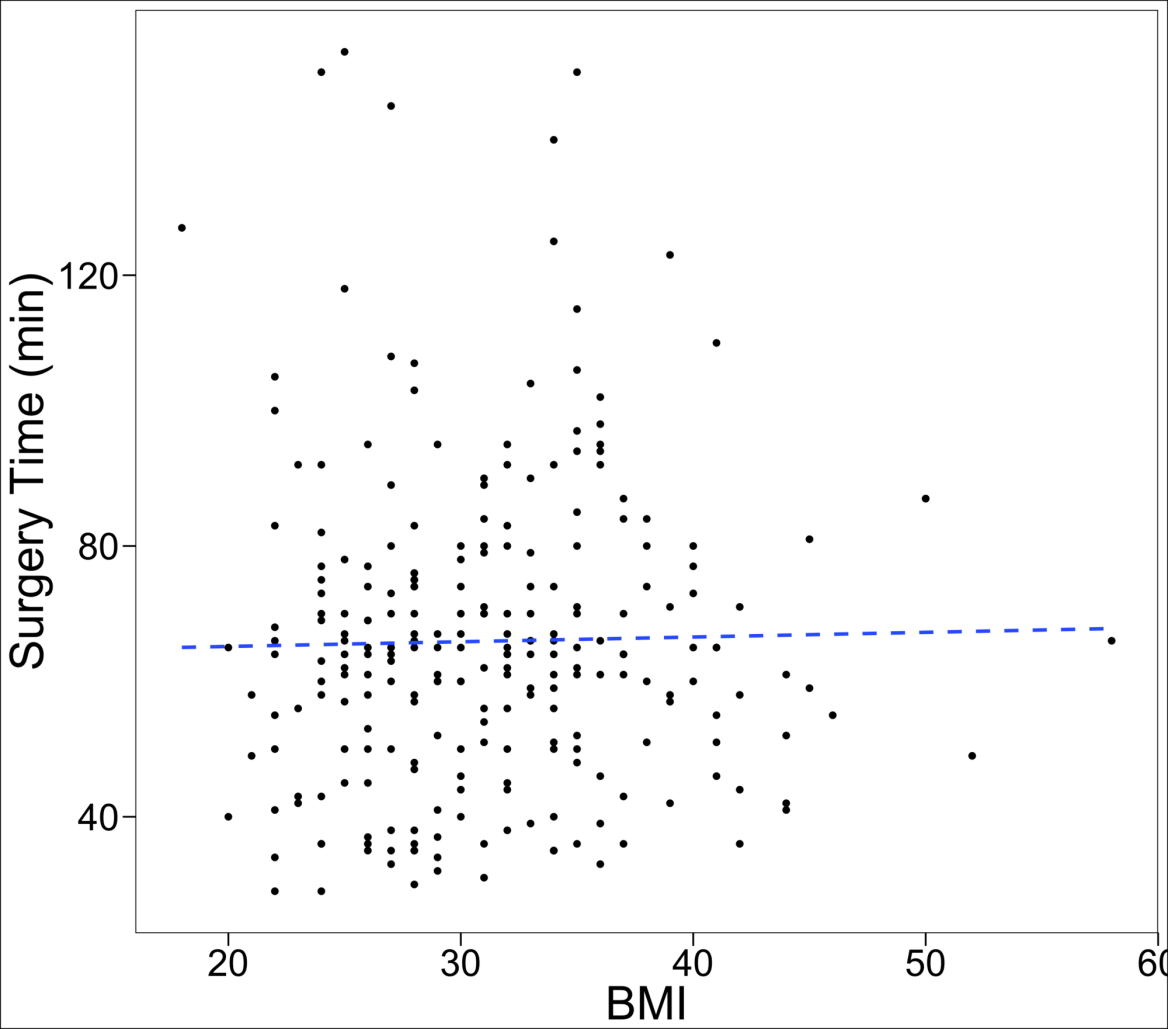
Relationship between surgery time and BMI for one- and two-level fusions. Linear regression line is plotted for reference. BMI: Body mass index

Perioperative outcomes are presented in Table 2, stratified by the number of surgical levels. For a single-level OLLIF, mean surgery time was 52 ± 18.9 minutes, with a blood loss of 42.2 ± 31.1 mL, 198.8 ± 87.2 seconds of fluoroscopy time and a hospital stay of 2.2 ± 1.7 days. Linear regression shows that controlling for the number of levels, there is no significant impact of BMI on surgery time (OLS coefficient 0.23, 95% CI -0.15 to 0.61) and that each additional level of surgery increases surgery time by 24.9 (95% CI 21.72 to 28.10) minutes. Figure 3 illustrates these results by plotting the relationship between surgery time and BMI for one- and two-level fusions.

**Table 2:** Perioperative outcomes stratified by the number of operative levels. All results are mean (SD). SD: Standard deviation.

Number of levels	1	2	3	4+
N	100	150	45	8
Surgery Time (min)	52.00 (18.94)	75.17 (20.77)	96.76 (21.75)	145.50 (46.72)
Blood Loss (mL)	42.24 (31.14)	63.34 (44.09)	110.53 (84.84)	151.50 (131.13)
Fluoroscopy Time (s)	198.94 (87.22)	315.84 (117.92)	431.53 (181.90)	645.38 (251.60)
Hospital Stay (days)	2.19 (1.70)	2.68 (1.43)	3.18 (1.27)	4.12 (1.96)

Complications are recorded in Table 3. There was one case of superficial wound infection, requiring five days of oral antibiotics and no further intervention. There was one case of postoperative bleeding leading to psoas compartment syndrome. The patient improved slowly and, at the one-year follow-up, exhibited 4/5 weakness in hip extension. Immediately after surgery, 22 patients (7.2%) met the criteria for nerve root irritation. At the one-year follow-up, six of those patients continued to meet the criteria for nerve root irritation, and the other 16 were asymptomatic. After surgery, 13 (4.3%) patients met criteria for neuropraxia. At the one-year follow-up, three of those patients were asymptomatic, nine met criteria for nerve root irritation and one patient continued to meet criteria for neuropraxia due to 3/5 tibialis anterior weakness. Amongst all 303 patients, 14 required reoperations within one year of the index surgery, including one patient with two reoperations. Indications for reoperation were screw fracture or loosening (n = 6), fall or motor vehicle accident (n = 5), and continued foraminal stenosis (n = 4).

**Table 3:** Perioperative and one-year complications. The one-year follow-up was at least 300 days after surgery. Amongst the 12 patients with nerve root irritation at the one-year follow-up, three patients had only a non-limiting sensory deficit. Amongst patients with reoperations, one patient had two reoperations; all other patients had one reoperation. MVA: Motor vehicle accident

Total number of patients (n)	303
Perioperative Complications	
Wound infection (n (%))	1 (0.3)
Bleeding (n (%))	1 (0.3)
Nerve Irritation (n (%))	22 (7.2)
Neuropraxia (n (%))	13 (4.3)
One-year Complications	
Patients seen for one-year follow-up (n)	204
Reoperation (n (%))	14 (4.6)
Screw Failure (n (%))	6 (2.0)
Fall or MVA (n (%))	5 (1.6)
Continued Stenosis (n (%))	4 (1.3)
Nerve Irritation (n (%))	15 (5.0)
Neuropraxia (n (%))	1 (0.3)

Patient-reported outcomes improved significantly by the one-year follow-up (Table 4). Ten-point pain scale improved from 8.6 ± 1.3 to 4.1 ± 3.0 (p < 0.001). Total ODI disability score improved from 56.6% ± 15.3% to 38.6% ± 21.4% (p < 0.001). Within the individual five-point scales on the ODI disability index, patients improved the most on the total pain intensity (1.7-point improvement, p < 0.001), followed by social life (1.1-point improvement, p < 0.001), work duties (0.95-point improvement, p < 0.001), standing (0.92-point improvement, p < 0.001) and sleeping (0.88-point improvement, p < 0.001). Patient improvement was least in the lifting (0.38-point improvement, p = 0.002), travel (0.67-point improvement, p < 0.001) and personal care (0.7-point improvement, p < 0.001) categories.

**Table 4:** Patient-reported outcomes at the one-year follow-up (at least 300 days post op). On the 10-point pain scale higher numbers represent higher levels of pain. The Oswestry is scored on a scale of zero to 100, with higher numbers representing increased disability. OP: Operation; SD: Standard deviation.

	Pre OP	Post OP	p
Ten-point pain scale (mean (SD)) {N}	8.6 ± 1.3 {297}	4.1 ± 3.0 {226}	<0.001
Total Oswestry Score (mean (SD)) {N}	56.59 (15.33) {239}	38.63 (21.40) {229}	<0.001
Pain Intensity (mean (SD))	3.90 (1.06)	2.16 (1.65)	<0.001
Personal Care (mean (SD))	2.13 (1.17)	1.44 (1.36)	<0.001
Lifting (mean (SD))	3.31 (1.18)	2.93 (1.32)	0.002
Walking (mean (SD))	2.86 (1.15)	1.99 (1.43)	<0.001
Sitting (mean (SD))	2.36 (1.30)	1.58 (1.29)	<0.001
Standing (mean (SD))	3.26 (1.21)	2.33 (1.54)	<0.001
Sleeping (mean (SD))	2.51 (1.29)	1.63 (1.33)	<0.001
Social Life (mean (SD))	2.91 (1.35)	1.81 (1.38)	<0.001
Travel (mean (SD))	2.20 (1.13)	1.53 (1.08)	<0.001
Work/House duties (mean (SD))	2.86 (1.13)	1.91 (1.24)	<0.001

**Figure 4:**
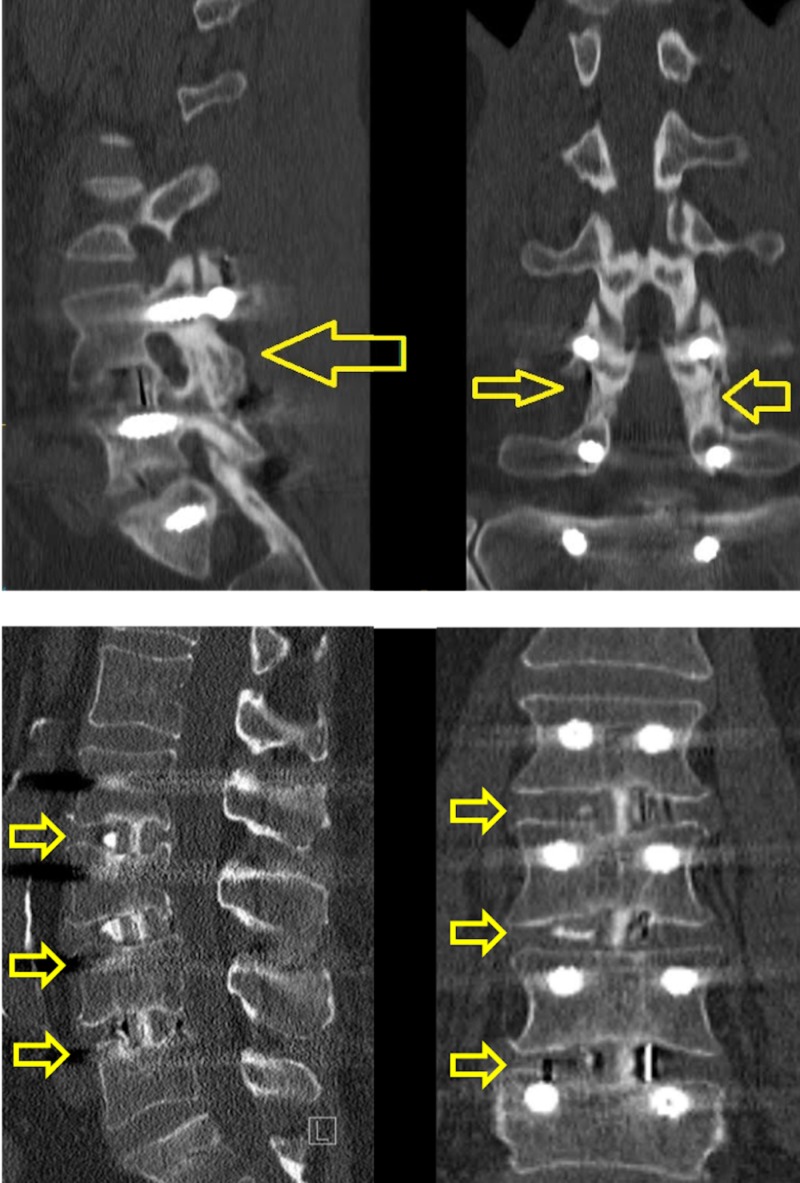
Representative CT scans one year after OLLIF surgery demonstrating (top) posterolateral fusion, (bottom) interbody fusion. Arrows indicate levels where fusion has occurred. CT: Computed tomography; OLLIF: Oblique lateral lumbar interbody fusion

Table 5 presents radiological outcomes. In our study group, 166 patients (54.8%) were imaged at least 300 days after surgery. These patients accounted for 307 surgical levels. Interbody fusion was achieved in 303 (98.7%) levels. Rates of posterolateral fusion were 69.1% and 66.4% on the right and left, respectively. Amongst patients for whom we acquired imaging one year after surgery, we placed 946 screws. There were 15 (1.6%) cases of screw fracture, 23 (2.4%) cases of screw loosening, 23 (2.4%) cases of screw breach and 32 (3.4%) cases of bicortical screw placement. Figure 4 shows representative CT scans showing posterolateral and interbody fusion.

**Table 5:** Radiographic outcomes at the one-year follow-up (≥300 days post-surgery).

Patients with imaging ≥300 days after surgery (n)	166
Total levels amongst patients with imaging (n)	307
Interbody Fusion (n (%))	303 (98.7)
Right Posterolateral Fusion (n (%))	212 (69.1)
Left Posterolateral Fusion (n (%))	204 (66.4)
Number of screws placed amongst patients with imaging (n)	946
Screw Fracture (n (%))	15 (1.6)
Screw Loosening (n (%))	23 (2.4)
Screw Breach (n (%))	23 (2.4)
Screw Bicortical (n (%))	32 (3.4)

## Discussion

In this study, we present the first evaluation of patient-reported and radiological outcomes delivered by OLLIF. Our series of 303 OLLIF operations on 568 surgical levels demonstrates that OLLIF can be routinely, safely and efficaciously used for lumbar spinal fusions from T12–L1 to L5–S1. This study is the first to report on clinical and radiological outcomes in OLLIF.

OLLIF is an extremely quick procedure, taking, on average, less than one hour (skin to skin) for a single-level procedure and an average of 75 minutes amongst all patients. This is one of the faster surgery times reported for spinal fusions in the literature. Other studies on MI fusions have cited surgery times between 104 and 390 minutes [8,19]. Typically, MIS lumbar fusions do not significantly reduce surgery time compared to open fusions [8]. OLLIF may be the first MI fusion that is faster than open procedures. We previously showed that OLLIF is faster than TLIF performed by the same surgeon, with a 69-minute decrease in surgery time for single-level procedures and larger differences for multilevel procedures [12]. In our experience, the OLLIF approach is extremely efficient because it avoids osteotomies, does not require direct visualization and the patient does not have to be repositioned during surgery like in other MI surgical procedures. Decreased surgery time is an important advantage because surgery and anesthesia time are amongst the more significant predictors of operative morbidity [20].

Like other MI surgeries, blood loss is low in OLLIF. A recent review found that MIS-TLIF surgeries typically reported blood loss between 51 mL and 496 mL, which is higher than the mean 42.2 mL blood loss in our single-level OLLIF group. We have previously shown that OLLIF reduces blood loss relative to TLIF, with a 321 mL reduction for a single-level procedure. Hospital stay is also typically lower in MI fusions compared to open equivalents. We have previously reported a 1.6-day reduction for OLLIF relative to TLIF [12]. In this study, single-level OLLIF patients stayed in the hospital for an average of 2.2 days, which is within the range of 1.8 to 11 days reported for other MI fusions [8]. Notably, six patients with single-level procedures and four patients with two-level procedures did not require overnight hospitalization and went home on the day of surgery. We anticipate being able to discharge many OLLIF patients faster than what was demonstrated in this study once the risk profile of OLLIF is clearly established.

Complications were extremely rare amongst our 303 patients. There was only one case (0.3%) of superficial wound infection that resolved with a brief course of oral antibiotics. Our infection rate was much lower than the infection rates reported in other studies of spinal fusions which typically range from 1.3% to 4.5% for MI fusions and 2.9% to 4.8% for open fusions [8,21,22]. OLLIF, when combined with pedicle screw fixation, requires multiple small incisions. However, the entire surgery is performed percutaneously without dissection, relying solely on gentle dilation of muscle and soft tissue. We hypothesize that the incidence of infection or abscess formation is low in OLLIF because the approach relies on gentle dilation of muscles. This reduces bleeding and prevents the formation of a cavity under the skin. Additionally, we avoid the use of electrocautery which creates necrotic tissue that can be a nidus for infection.

Nerve damage is one of the major complications associated with lumbar fusions, and there is particular concern regarding this complication in MI procedures, where neural structures are not directly visualized. At the one-year follow-up, 15 (5%) patients had nerve irritation, and only one patient (0.3%) had neuropraxia. Given the lack of standardized definitions regarding nerve injury, rates may be difficult to compare between different sources. Broadly, these rates are comparable to rates of nerve deficits in MI-TLIF [8] but lower than in LLIF where rates of nerve injury range from 6.1% to 11.9% [23,24]. In our experience, most patients with persistent nerve irritation experience mild paresthesias in the L5 distribution that does not limit their activity or cause significant discomfort. Previous studies that have not used neurophysiological monitoring have found high complication rates with an approach through Kambin’s triangle [14]. Our data and clinical experience suggest that neurophysiological monitoring is sufficient to ensure a safe approach during the OLLIF procedures. A recent study demonstrates that if neurophysiological safety thresholds are not met, OLLIF can be salvaged with endoscopic foraminotomy [25].

This is the first study to report that patient-reported outcomes measured by a ten-point pain scale and ODI improved significantly after OLLIF. In a review of 12 studies on MI lumbar fusions where the ODI was measured [8], patients improved between 10.7 and 33 points, with a median of 16 points. In our study, patients improved by 18 points on the ODI. It should be noted the patients in our study had an average ODI score of 57 prior to surgery, which is higher than in most other MI fusion studies [19]. The reason for these higher preoperative ODI scores may be that we apply strict protocols and checklists for conservative therapy before patients are considered surgical candidates.

Several patients in this study had been turned down for surgery by other surgeons due to comorbidities such as severe obesity, advanced age and other medical conditions. This study includes 32 patients with Class III obesity with a maximum BMI of 58 kg/m^2^. We demonstrate that in OLLIF, increased BMI has no effect on surgery time. In contrast, we have previously shown that surgery time is directly correlated with BMI in MIS-TLIF and open TLIF. In open procedures, more time is spent dissecting and closing if the patient is obese. In OLLIF, however, no dissection of soft tissue is required. The probe that is used to gain access to the disc space can be advanced quickly through the subcutaneous layers of fat, muscle and fascia. In our experience, the difficulty of OLLIF does not increase with obesity.

Radiographic outcomes showed that interbody fusion was achieved in 98.7% of imaged levels in our study. While this figure is high, it is consistent with other studies of minimally invasive fusions. In two meta-analyses of fusion rates in minimally invasive lumbar fusions, fusion rates were between 94.8% and 97.1% [8,26]. The most important factor impacting fusion rates is that in OLLIF, unlike in open surgery, the posterior aspect of the spine is not stripped of muscle. The majority of the blood supply to the posterior spinal column arrives through the paraspinal muscles [27]. We hypothesize that preservation of paraspinal muscles and blood supply to the vertebral column also leads to lower rates of adjacent level disease in OLLIF compared to open fusions; we are currently collecting data to investigate this. Another factor allowing for improved fusion in OLLIF is the surgeon can freely pack the disc space with tricalcium phosphate because the opening of the disc space is small and sealed by the cage, reducing the risk of leak and subsequent compression of neural structures. We have observed radiographic evidence of fusion in OLLIF patients as early as four months postoperatively when CT scans are obtained for other reasons. We are currently designing a study to evaluate how fast fusion is typically achieved in OLLIF.

In our experience, the conical tip of the cage used in OLLIF allows us to place cages that are, on average, 3 mm taller than in TLIF. This taller cage increases the intervertebral distance and provides foraminal decompression without foraminotomy, an effect we call physiologic decompression. In the present study, only four patients (1.3%) underwent reoperation for continued foraminal stenosis. Due to the increased cage size, we have even been able to achieve correction of spinal deformity by strategically adjusting the cage placement [16].

There is mixed evidence on the cost of MI surgeries relative to open procedures. Some studies find small cost increases driven mostly by the cost of implants and devices [28], while others report cost savings driven by lower postoperative cost and faster return to work [29]. We have previously shown that perioperative costs associated with operating room time and hospital stay are substantially reduced by OLLIF relative to TLIF and can result in savings of up to $14,240 per procedure [30]. The present study shows that the perioperative outcomes we reported previously can be reproduced over a larger cohort. This evidence implies that the perioperative savings projected in our earlier study also apply to this larger cohort. A detailed economic analysis is currently underway.

There are several limitations to this study. This study is a retrospective analysis, and our patient population is predominantly from rural areas, making consistent follow-up challenging. In our experience, patients most commonly refuse to return for follow-up visits if they are doing well and do not perceive a need to return to our clinic. We are planning to compare the clinical outcomes of those patients who participate in regular follow-up visits to those patients that we must contact for patient-reported outcomes. Additionally, as this is a single-surgeon study, a multi-center study is needed to ensure external validity of our results. Finally, we present evidence on one type of procedure only. While we have previously compared OLLIF to TLIF and showed that OLLIF significantly improves perioperative outcomes relative to TLIF [12], our data do not rise to Class I evidence, which would require a randomized controlled trial.

## Conclusions

This study is the first to present outcomes in a large cohort of OLLIF patients. We demonstrate that OLLIF is a safe, efficient and efficacious technique for fusions of the lumbar spine from T12-L1 to L5-S1. In OLLIF, the spine is approached without compromising supportive connective tissue, muscles or osseous structures. This allows for faster surgeries and short hospitalization even in patients with significant disability and obesity. Based on our perioperative, clinical, and radiographic data we propose that OLLIF should be considered a preferred option for fusions of the lumbar spine.

## References

[1] Andersson GBJ (1999). Epidemiological features of chronic low-back pain. Lancet.

[2] Kirkaldy-Willis WH, Wedge JH, Yong-Hing K, Reilly J (1978). Pathology and pathogenesis of lumbar spondylosis and stenosis. Spine.

[3] Luo X, Pietrobon R, Sun SX, Liu GG, Hey L (2004). Estimates and patterns of direct health care expenditures among individuals with back pain in the United States. Spine.

[4] Cloward RB (1953). The treatment of ruptured lumbar intervertebral discs by vertebral body fusion. I. Indications, operative technique, after care. J Neurosurg.

[5] Humphreys SC, Hodges SD, Patwardhan AG, Eck JC, Murphy RB, Covington LA (2001). Comparison of posterior and transforaminal approaches to lumbar interbody fusion. Spine.

[6] Sihvonen T, Herno A, Paljärvi L, Airaksinen O, Partanen J, Tapaninaho A (1993). Local denervation atrophy of paraspinal muscles in postoperative failed back syndrome. Spine.

[7] Ozgur BM, Aryan HE, Pimenta L, Taylor WR (2006). Extreme lateral interbody fusion (XLIF): a novel surgical technique for anterior lumbar interbody fusion. Spine J.

[8] Goldstein C, Macwan K, Sundararajan K, Rampersaud YR (2014). Comparative outcomes of minimally invasive surgery for posterior lumbar fusion: a systematic review. Clin Orthop Relat Res.

[9] Mayer MH (1997). A new microsurgical technique for minimally invasive anterior lumbar interbody fusion. Spine.

[10] Quillo-Olvera J, Lin GX, Jo HJ, Kim JS (2018). Complications on minimally invasive oblique lumbar interbody fusion at L2-L5 levels: a review of the literature and surgical strategies. Ann Transl Med.

[11] Arnold PM, Anderson KK, McGuire RA (2012). The lateral transpsoas approach to the lumbar and thoracic spine: a review. Surg Neurol Int.

[12] Abbasi HR, Abbasi AB (2015). Oblique lateral lumbar interbody fusion (OLLIF): technical notes and early results of a single surgeon comparative study. Cureus.

[13] Kambin P (1996). Arthroscopic lumbar intervertebral fusion. The Adult Spine: Principles and Practice.

[14] Jacquot F, Gastambide D (2013). Percutaneous endoscopic transforaminal lumbar interbody fusion: is it worth it?. Int Orthop.

[15] Abbasi HR, Abbasi AB (2017). Minimally invasive direct lateral interbody fusion (MIS-DLIF): proof of concept and perioperative results. Cureus.

[16] Abbasi HR, Miller L, Abbasi AB, Orandi V, Khaghany K (2017). Minimally invasive scoliosis surgery with oblique lateral lumbar interbody fusion: single surgeon feasibility study. Cureus.

[17] Foley KT, Gupta SK, Justis JR, Sherman MC (2001). Percutaneous pedicle screw fixation of the lumbar spine. Neurosurg Focus.

[18] Karikari IO, Isaacs RE (2010). Minimally invasive transforaminal lumbar interbody fusion: a review of techniques and outcomes. Spine.

[19] Turrentine FE, Wang H, Simpson VB, Jones RS (2006). Surgical risk factors, morbidity, and mortality in elderly patients. J Am Coll Surg.

[20] Smith JS, Shaffrey CI, Sansur CA (2009). Rates of infection after spine surgery based on 108,419 procedures: 925. Neurosurgery.

[21] McGirt MJ, Parker SL, Lerner J, Engelhart L, Knight T, Wang MY (2011). Comparative analysis of perioperative surgical site infection after minimally invasive versus open posterior/transforaminal lumbar interbody fusion: analysis of hospital billing and discharge data from 5170 patients. J Neurosurg Spine.

[22] Fairbank JC, Pynsent PB (2000). The Oswestry disability index. Spine (Phila Pa 1976).

[23] Pumberger M, Hughes AP, Huang RR, Sama AA, Cammisa FP, Girardi FP (2012). Neurologic deficit following lateral lumbar interbody fusion. Eur Spine J.

[24] Lykissas MG, Aichmair A, Hughes AP (2014). Nerve injury after lateral lumbar interbody fusion: a review of 919 treated levels with identification of risk factors. Spine J.

[25] Katzell J (2014). Endoscopic foraminal decompression preceding oblique lateral lumbar interbody fusion to decrease the incidence of post operative dysaesthesia. Int J Spine Surg.

[26] Wu RH, Fraser JF, Härtl R (2010). Minimal access versus open transforaminal lumbar interbody fusion: meta-analysis of fusion rates. Spine (Phila Pa 1976).

[27] Crock HV, Yoshizawa H (1977). The Blood Supply of the Lumbar Vertebral Column. Clin Orthop Relat Res.

[28] Twitchell S, Karsy M, Reese J, Guan J, Couldwell WT, Dailey A, Bisson EF (2018). Assessment of cost drivers and cost variation for lumbar interbody fusion procedures using the Value Driven Outcomes database. Neurosurg Focus.

[29] Parker SL, Adogwa O, Bydon A, Cheng J, McGirt MJ (2012). Cost-effectiveness of minimally invasive versus open transforaminal lumbar interbody fusion for degenerative spondylolisthesis associated low-back and leg pain over two years. World Neurosurg.

[30] Abbasi HR, Murphy CM (2015). Economic performance of oblique lateral lumbar interbody fusion (OLLIF) with a focus on hospital throughput efficiency. Cureus.

